# HER2 Ile655Val Single Nucleotide Polymorphism in Patients with Ovarian Cancer

**DOI:** 10.5812/ircmj.2173

**Published:** 2013-01-05

**Authors:** Zahra Mojtahedi, Nasroolah Erfani, Mahyar Malekzadeh, Mohammad Reza Haghshenas, Abbas Ghaderi, Alamtaj Samsami Dehaghani

**Affiliations:** 1 Shiraz Institute for Cancer Research, Shiraz University of Medical Sciences, Shiraz, IR Iran; 2 Department of Obstetrics and Gynecology, Shiraz University of Medical Sciences, Shiraz, IR Iran

**Keywords:** Carcinoma, Endometrioid, HER2, Polymorphism, Single Nucleotide

## Abstract

**Background:**

The association between HER2 Ile655Val single nucleotide polymorphism and cancer is controversial.

**Objectives:**

The aim of our study was to investigate this polymorphism in patients with ovarian cancer.

**Patients and Methods:**

Genomic DNA was extracted from peripheral blood leukocytes of 107 patients and 130 healthy women. HER2 gene polymorphism was assessed by PCR-RFLP.

**Results:**

No significant difference was observed in genotype and allele frequency between patient and control groups according to HER2 Ile655Val polymorphism. The disease stage, age, and histological type were also not associated with the polymorphism.

**Conclusions:**

Our data showed that HER2 Ile655Val single nucleotide polymorphism was not significantly associated with onset, histological type, age, and stage of ovarian cancer in Iranian patients.

## 1. Background

Ovarian cancer is the sixth most common cancer among women. It is estimated that 190,000 new cases and 114,000 deaths occur due to this malignancy each year worldwide (1). The majority of patients are diagnosed at a late clinical stage due to absence of early symptoms. Epithelial ovarian carcinoma, sex-cord stromal tumors, and germ-cell tumors are three major types of ovarian cancer, of which, the former is the most common type (2). HER2, similar to the other four members of epidermal growth factor receptor (EGFR), possesses an extracellular ligand-binding domain, a single hydrophobic trans-membrane region, and an intracellular tyrosine kinase domain (3). HER2 is activated by the formation of heterodimers with other members of EGFR family, and involves in the growth of both normal tissue and malignant tumors (3). HER2 is well-known for its role in breast cancer. Its over-expression occurs in approximately 20% of patients with breast cancer, which is correlated with poor prognosis (3, 4). Over-expression of HER2 was also reported in ovarian cancer, although to a lesser extent than that in breast cancer (5). A point mutation altering the membrane spanning region of the HER2 gene transforms the normal gene to a potent oncogene in an animal model (6) In human HER2 gene, a single nucleotide polymorphism was identified in the trans-membrane coding region at codon 655 that encodes either isoleucine (ATC) or valine (GTC). It has been shown that valine-expressing cells display a higher growth rate compared to isoleucine-expressing cells (7). However, the association between HER2 Ile655Val polymorphism with cancer is controversial (8-11).

## 2. Objectives

The aim of the present case-control study was to compare HER2 Ile655Val genotype and allele distributions between patients with ovarian cancer and healthy individuals. The association between HER2 Ile655Val polymorphism and histological type, age, and tumor stage at diagnosis was also investigated.

## 3. Patients and Methods

### 3.1. Subjects

The study was approved by the Ethics Committee of Shiraz University of Medical Sciences. Samples were drawn from each participant after informed consent. A total number of 107 non-relative patients who were operated between 2005 and 2010 at Faghihi or Zeinabieh Hospital, Shiraz, Iran, were enrolled for genotyping. Diagnosis of ovarian cancer was confirmed histopathologically. Seventy-seven patients suffered from epithelial and 30 from sex-cord stromal and germ-cell tumors. The characteristics of patients were obtained from patients' files. The mean age of patients at diagnosis was 45.9 ± 16.1 years. The stage of disease was determined according to the International Federation of Gynecology and Obstetrics (FIGO) staging. Control group was comprised of 130 healthy women with mean age of 46.6 ± 15.5 years. They had no history of cancer, an autoimmune, or serious infectious disease.

### 3.2. HER2 Polymorphism

Venus peripheral blood samples (10 mL) were collected and genomic DNA was extracted by the salting out method (12). HER2 Ile655Val polymorphism was investigated by PCR-restriction fragment length polymorphism (PCR-RFLP) as previously described by Xie et al. (11). The resultant 148 bp products were digested by BsmAI (Fermentas, Lithuania) and analyzed on 3% agarose gels. The presence of Val allele of HER2 gene was identified by 116 and 32 bp fragments, and Ile allele gave a single 148 bp product.

### 3.3. Statistical Analysis 

The data were analyzed using SPSS software (version 11.5.0; SPSS, Chicago, IL, USA). Pearson's chi-square test was used to compare differences in genotypes and alleles between patients and controls, epithelial and sex-cord stromal/germ-cell tumors, and stage I-II and stage III-IIII of the disease. The difference in age at onset according to the HER2 polymorphism was calculated by one-way analysis of variance (ANOVA). Findings were considered statistically significant at a p value less than 0.05.

## 4. Results

HER2 Ile655Val genotypes and alleles in 107 patients with ovarian cancer were compared with those in 130 healthy age-matched controls (Table 1). There was no association between total patients with ovarian cancer and healthy controls in HER2 genotypes (P = 0.56) and alleles (P = 0.69). Subdivision of patients revealed a P value of 0.86 for genotype, and of 0.66 for allele differences between healthy individuals and epithelial ovarian cancer, and a P value of 0.66 and 0.91 for genotype and allele differences, respectively, between healthy individuals and sex-cord stromal/ germ-cell tumors (Table 1). Moreover, the difference in genotypes and alleles between epithelial tumor and sex-cord stromal/ germ-cell tumors showed a P = 0.78 for genotypes and P = 0.84 for alleles. The data regarding the stage of disease at diagnosis was available for 49 patients, of which, 15 patients (30.6%) were in early stage of I-II and 34 patients (69.4%) in late stage of III-IV. As for genotypes, 11 (73.3%) and 4 patients (26.7%) in stage I-II of the disease showed Ile/Ile and Ile/Val genotypes, respectively. In stage III-IV of the disease, 28 (82.3%) and 6 (17.7%) patients had Ile/Ile genotype, and Ile/Val or Val/Val genotypes, respectively (P = 0.47 for genotype differences in stage I-II vs. stage III-IV). The frequency of Ile allele was 26/30 (86.7%) and 61/68 (89.7%) in stage I-II and stage III-IV, respectively. The Val frequency was 4/30 (13.3%) in stage I-II, and 7/68 (10.3%) in stage III-IV (P = 0.66 for allele differences in stage I-II vs. stage of III-IV). Mean age of onset of ovarian cancer did not differ in patients according to the HER2 polymorphism (P = 0.68). The mean age of patients with Ile/Ile genotype was 45.9 ± 17.5 years, with Ile/Val genotype was 45.3 ± 12.3 years, and with Val/Val genotype (1 patient) was 60 years.

**Table 1:** Allele and Genotype Frequencies of HER2 Gene in 107 Patients With Ovarian Cancer and 130 Controls

	Total tumors (n = 107) a	Epithelial type (n = 77)	Sex-cord/germ-cell (n = 30)	Healthy controls (n = 130)
**Genotype frequency, No. (%)**				
Ile/Ile	81 (75.7)	59 (76.6)	22 (73.3)	97 (74.6)
Ile/Val	25 (23.4)	17 (22.1)	8 (26.7)	30 (23.0)
** Val/ Val**	1 (1.0)	1 (1.3)	0 (0.0)	3 (2.4)
**Allele frequency, No. (%)**				
Ile	187/214 (87.4)	135/154 (87.7)	52/60 (86.7)	224/260 (86.1)
Val	27/214 (12.6)	19/154 (12.3)	8/60 (13.3)	36/260 (13.9)

^a^P values calculated using chi-square test were not significant

## 5. Discussion

Ovarian cancer is the major cause of death due to gynecological cancers (1). Identification of novel biomarkers with application in diagnosis, prognosis, or therapy is a high priority in ovarian cancer (2). The HER2 over-expression was initially noted in breast cancer (approximately 20% of cases) (3). Further studies also showed HER2 involvement in the pathogenesis of ovarian cancer, although to a lesser extent than that in breast cancer (5). A point mutation altering trans-membrane region of counterpart of human HER2 gene transforms normal gene to a potent oncogene in animal models (6). However, the association between Ile655Val polymorphism in trans-membrane region of human HER2 gene, which is possibly functional, and breast cancer, is conflicting. Some research studies and meta-analyses indicated significant association of this polymorphism with breast cancer onset, age at diagnosis, or stage of the disease, but other research studies and meta-analyses rejected these associations (8, 9, 11). A few data are available regarding the association of HER2 Ile655Val polymorphism with ovarian cancer. Pinto et al. found no significant differences in distributions of HER2 Ile655Val genotypes and alleles between patients with ovarian cancer and healthy individuals in Portugal. Moreover, they found no association between this polymorphism and age, stage, and histological type of ovarian cancer. However, they indicated that patients carrying the valine homozygotic genotype had a lower overall mean of survival (10). We investigated HER2 Ile655Val polymorphism in patients with ovarian cancer and compared them with those in a healthy control group. We also found no significant association between HER2 Ile655Val genotypes or alleles and ovarian cancer onset, histological types, age, and stage of the disease. The absence of association between germ-line HER2 Ile655Val polymorphism and ovarian cancer does not decline the importance of Val versus Ile alleles in the cancer. In fact, an allelic imbalance was reportedly found in both breast and ovarian malignant tissues. For example, Puputti et al. found an allelic imbalance in breast tumor tissues with over-representation of Val allele. In contrast to breast tumors, Ile allele was over-represented in ovarian cancer (13). In conclusion, HER2 Ile655Val polymorphism is unlikely to affect ovarian cancer onset, histological type, age, and stage of the disease in Iranian patients. Further studies are necessary to elucidate functional significance of allelic imbalance of Val versus Ile alleles in ovarian cancer tissues.
